# The common functional *FKBP5* variant rs1360780 is associated with altered cognitive function in aged individuals

**DOI:** 10.1038/srep06696

**Published:** 2014-10-21

**Authors:** Takashi Fujii, Miho Ota, Hiroaki Hori, Kotaro Hattori, Toshiya Teraishi, Junko Matsuo, Yukiko Kinoshita, Ikki Ishida, Anna Nagashima, Hiroshi Kunugi

**Affiliations:** 1 Department of Mental Disorder Research, National Institute of Neuroscience, National Center of Neurology and Psychiatry, 4-1-1, Ogawahigashi, Kodaira, Tokyo, 187-8502, Japan

## Abstract

The common single nucleotide polymorphism (SNP) rs1360780 (C/T) of the FK506 Binding Protein 5 (*FKBP5*) gene has been reported to be associated with an altered response of the hypothalamic-pituitary-adrenal (HPA) axis and the development of stress-related psychiatric disorders such as posttraumatic stress disorder (PTSD). In the present study, we examined whether this SNP is associated with cognitive function in a non-clinical population. The full versions of the Wechsler Memory Scale-Revised and Wechsler Adult Intelligence Scale-Revised were administered to 742 and 627 Japanese individuals, respectively, followed by genotyping of rs1360780 by the TaqMan 5′-exonuclease allelic discrimination assay. For both cognitive tests, we found significantly poorer attention/concentration (working memory) in aged (>50 years old) individuals carrying the T allele compared with their counterparts. This finding accords with an altered HPA axis and vulnerability to stress-related psychiatric disorders.

FK506 Binding Protein 5 (FKBP5) is a key molecule in the stress response and the pathophysiology of stress-related disorders including post-traumatic stress disorder (PTSD) and major depressive disorder (MDD)^1^,^2^. An altered stress response in hypothalamic- pituitary- adrenal (HPA) axis reactivity has been implicated in these disorders^3^,^4^,^5^. In the HPA axis, FKBP5 plays a role as a glucocorticoid receptor (GR)-regulating co-chaperone molecule of heat shock protein 90 by binding to GRs in the cytosol and decreasing GR nuclear translocation^1^. FKBP5 thereby inhibits the function of GRs which regulate adrenocortical secretion of glucocorticoids (cortisol in humans and corticosterone in rodents) during stress-induced HPA axis activity.

To our knowledge, *FKBP5* rs1360780 (C/T) is the only common single nucleotide polymorphism (SNP) among *FKBP5* polymorphisms that has been demonstrated to have functional effects, despite being located within intron 2. The association between this SNP and FKBP5 protein expression levels has been well established. The T allele has been considered a high induction allele for *FKBP5* by cortisol when compared with the C allele^6^. The sequence containing the T allele of this SNP forms a putative TATA box, and exhibits stronger binding activity to the TATA box binding protein when compared with the C allele. These molecular changes lead to alteration of the chromatin interaction between the *FKBP5* transcription start site and long-range enhancer, and results in the enhancement of *FKBP5* mRNA transcription^7^. Accumulating evidence suggests that the T allele is a risk factor in early/childhood trauma, and predicts PTSD^8^,^9^, suicide attempts^10^, MDD^11^,^12^, and current PTSD symptoms^7^. In the recent study^13^, FKBP5 levels in the human brain were reported to be associated with Alzheimer's disease (AD) progression although there is no genetic association study between *FKBP5* rs1360780 and AD.

Recently, we found that, in the aged (>50 years old) non-clinical population, individuals carrying the T allele of rs1360780 showed lower cortisol reactivity to the dexamethasone/corticotropin-releasing hormone (DEX/CRH) test than non-T carriers^2^. Furthermore, aged T carriers showed significantly higher and lower mRNA expression levels of *GR* and *FKBP5* in peripheral blood mononuclear cells, respectively, when compared with aged non-T carriers^2^. Interestingly, these biochemical phenotypes of the aged non-clinical subjects carrying the risk T allele accords with the endophenotypes reported in patients with PTSD^4^,^14^.

Patients with PTSD are known to have difficulty in concentrating^15^ and exhibit mild cognitive deficits^16^. HPA axis reactivity contributes to normal cognitive function^17^. To our knowledge, however, there is no information in the literature on the genetic effects of *FKBP5* on neurocognitive functions.

In the present study, we examined the possible association between *FKBP5* rs1360780 and neurocognitive function. We hypothesized that even non-clinical individuals carrying the T allele would show impaired cognitive function. Because the above-mentioned association of rs1360780 with HPA axis reactivity was observed in an age-dependent manner in our previous study^2^, we took age into account in this present study.

## Results

### Demographic characteristics of non-clinical subjects

Neurocognitive performance was assessed using the Wechsler Memory Scale-Revised (WMS-R) and the Wechsler Adult Intelligence Scale-Revised (WAIS-R) in 743 and 627 subjects, respectively. Since the genotype frequency of homozygotes for the T allele (T/T) was small (0.044), those individuals homozygous and heterozygous for the T allele were combined in the analysis. There was no significant difference in mean age, education years, or gender distribution between the genotype groups (CC vs. CT/TT; Table 1). The genotype distribution for subjects administered either the WMS-R or WAIS-R did not significantly deviate from the Hardy-Weinberg equilibrium (HWE) (*P* > 0.05).

### Aged T carriers showed poorer attention/concentration on the WMS-R

**Figure 1:**
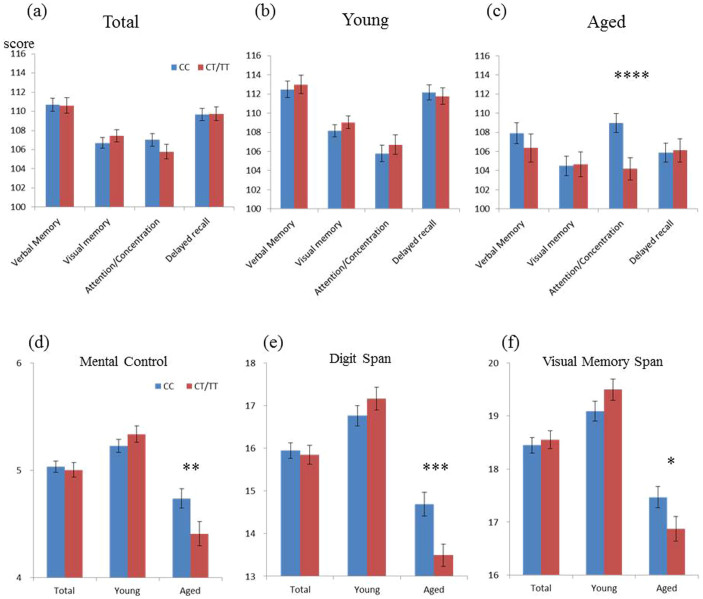
Mean scores on the WMS-R tests for both genotypes (CC and CT/TT) in the total, aged, and young groups. No significant differences by genotype were observed in the total (a: CC [*n* = 423], CT/TT [*n* = 320]) or young (b: CC [*n* = 256], CT/TT [*n* = 205]) groups. We found a significant genotype difference in mean attention/concentration scores on the WMS-R in the aged group (c; *F* = 14.94, *df* = 1, **** *P* = 0.00014: CC [*n* = 167], CT/TT [*n* = 115]). No significant difference in any WMS-R attention/concentration subtests ([d] mental control, [e] digit span, [f] visual memory span) between the two groups (CC vs. CT/TT) was observed in the total or young groups. In the aged group, T carriers show statistically lower mean scores in all subtests ([d] mental control, *F* = 6.98, *df* = 1, **P = 0.0087; [e] digit span, *F* = 8.36, *df* = 1, ***P = 0.0041; [f] visual memory span, *F* = 4.60, *df* = 1, *P = 0.033), than non-T carriers. Error bars indicate standard error of the mean (S.E.M).

Across all subjects, there was no significant difference in the WMS-R between the two genotype groups (Figure 1a). When young (≤50 years old) and aged groups were examined separately, as in our previous study^2^, the mean score of attention/concentration was significantly lower in T than in non-T carriers in the aged group (*F* = 14.9, *df* = 1, *P* = 0.00014; Figure 1c), but not in the young group (Figure 1b). The result remains significance even after the Bonferroni correction (critical *P* = 0.05/8 = 0.00625 due to 4 cognitive domains for 2 age groups). There was no significant difference in the other categories (verbal memory, visual memory, or delayed recall) between the genotype groups, even in the aged subjects (Figure 1a–c). Relationships between the genotype and WMS-R attention/concentration subtests are shown in Figure 1d–f. In the aged group, compared to non-T carriers, T carriers showed statistically lower mean scores on all subtests (mental control, *F* = 7.0, *df* = 1, *P* = 0.0087; digit span, *F* = 8.4, *df* = 1, *P* = 0.0041; visual span, *F* = 4.6, *df* = 1, *P* = 0.033). Although 3 subtests comprising WMS-R attention/concentration index were examined in young and aged groups, the main effect for digit span was significant in the aged group even after the Bonferroni correction (critical *P* < 0.05/6 = 0.0083).

### Aged T carriers had lower digit span scores on the WAIS-R

**Figure 2:**
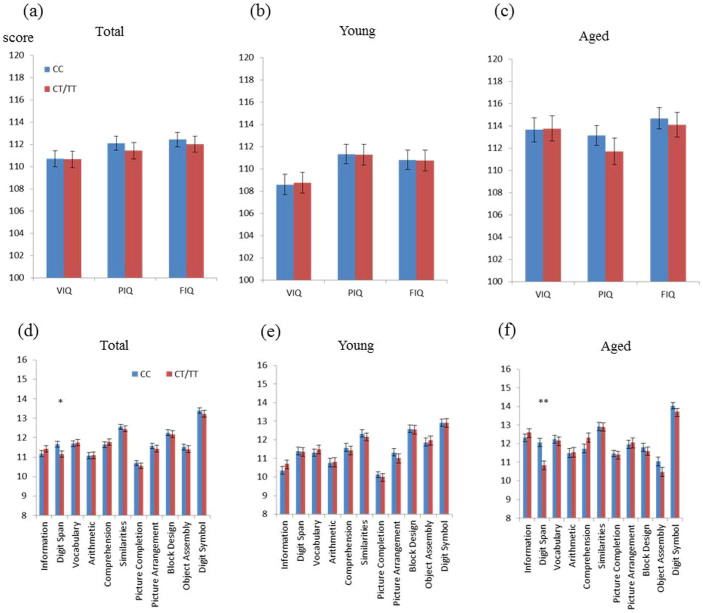
Mean scores from IQ and each subset in the verbal and performance sections of the WAIS-R in the total, aged, and young groups. Mean IQ scores assessed using the WAIS-R in T and non-T carriers are shown for the total (a: CC [*n* = 360], CT/TT [*n* = 267]), young (b: CC [*n* = 210], CT/TT [*n* = 165]), and aged (c: CC [*n* = 150], CT/TT [*n* = 102]) groups. There was no significant difference in verbal IQ (VIQ), performance IQ (PIQ), or full-scale IQ (FIQ) between genotypes (CC vs. CT/TT) from any group (total, aged, or young). Error bars indicate S.E.M. A significant difference in digit span by genotype in the total (d) and aged (f) groups was observed using the WAIS-R. The young group (e) showed no significant differences in any of the subsets. Error bars indicate S.E.M. * *P* = 0.0060, ** *P* = 0.00020 (ANCOVA).

With respect to the WAIS-R, there was no significant difference between the two genotype groups in full-scale Intelligence Quotient (IQ), verbal IQ, or performance IQ in the total (Figure 2a), aged (Figure 2b), or young (Figure 2c) groups. Relationships between the genotype of *FKBP5* rs1360780 and WAIS-R subscales in the total, young, and aged groups are shown in Figure 2d–f, respectively. In the total group, the mean score on the digit span subtest of the WAIS-R was significantly lower in T carriers than in non-T carriers (*F* = 7.6, *df* = 1, *P* = 0.0060; Figure 2d). In the young group, there was no significant difference in any subscale scores between the two genotype groups (Figure 2e). In contrast, the mean score on the digit span subtest in the WAIS-R was significantly lower in the aged T carriers than in the aged non-T carriers (*F* = 14.2, *df* = 1, *P* = 0.00020; Figure 2f), as expected from the WMS-R results. Although 11 subtests in the WAIS were examined in young and aged groups, the main effect for digit span was significant in the aged group even after the Bonferroni correction (critical *P* < 0.05/22 = 0.0023).

## Discussion

We found that the *FKBP5* functional polymorphism rs1360780 was associated with novel phenotypes in cognitive function, even in a non-clinical population. In accordance with our previous study^2^ on the relationship between this SNP and HPA axis reactivity, an age-dependent effect was observed. Aged subjects with the T allele had a significantly lower mean score on the attention/concentration index in the WMS-R compared with their, non-T, aged counterparts. In particular, they showed poor performance on the digit span subtest of the WMS-R. Similar results were obtained when cognitive functions were assessed by the WAIS-R.

FKBP5 is responsive to stressor exposure and modulates GR sensitivity^1^. *FKBP5* expression was reported to be upregulated in many brain regions after exposure to stress^18^. However, there are differences in GR sensitivity among brain regions. For example, the baseline level of *FKBP5* expression is higher in the hippocampus than in other brain regions^19^, and thereby extreme stressor exposure is needed to increase *FKBP5* levels in the hippocampus^18^. Such differences in *FKBP5* function among brain regions may create the *FKBP5* genotype-dependent vulnerability in some specific brain regions, thereby likely leading to the difference in cognitive function between the genotypes of rs1360780.

Attention/concentration impairment is considered one of the symptoms of PTSD^16^. PTSD is characterized by 4 primary symptoms, namely intrusion, numbing, avoidance, and impairment of arousal. Impaired attention/concentration is included within the arousal symptom^15^. According to a previous review^16^, 16 out of 19 studies on neurocognitive functioning in PTSD provided evidence of attention/immediate memory deficits. In a more recent study using the WMS-R, patients with PTSD were significantly impaired in the attention/concentration index when compared with trauma-exposed non-PTSD volunteers^19^. Overall, accumulating evidence supports the mild impairment of attention and immediate memory in PTSD patients. Given the results of the present study in a non-clinical population, poor attention/concentration may be an endophenotype of individuals who have a genetic risk (i.e., the T allele of rs1360780) for PTSD rather than the result of the development of the illness.

Attention/concentration impairment is common in MDD^20^. In homozygotes for the T allele of rs1360780, trauma exposure during childhood and adolescence increased the risk of developing depression^12^. Patients with AD also show global cognitive impairment including decreased attention and concentration^21^. Thus, the phenotypes of aged T carriers in the present study may be similar to the endophenotypes of not only patients with PTSD but also patients with MDD or AD.

Our previous study showed that aged non-clinical individuals carrying the T allele of rs1360780 had a suppressed cortisol response in the HPA axis^2^. However, previous studies in aged populations reported conflicting results regarding the association between cortisol levels and cognitive performance^22^,^23^,^24^,^25^,^26^,^27^. Although, compared with the C allele, the T allele is associated with higher FKBP5 induction by cortisol^6^,^7^, aged T carriers exhibit low cortisol levels likely due to compensatory mechanisms that occur during aging^2^,^28^. The relationship between *FKBP5* genotype, cortisol, and cognitive performance is complex and further investigations are required to understand its underpinning molecular mechanisms. However, we could provide a possible mechanism. We previously found lower HPA axis reactivity in T carriers compared with non-T carriers in the aged population^2^. This lower reactivity was supported by the increased *GR* and reduced *FKBP5* expression levels in aged T carriers' peripheral blood mononuclear cells (PBMCs)^2^. Such changes could extend beyond PBMCs to the brain and be involved in the structural changes in specific brain regions. In our previous study, T carriers exhibited the smaller dorsal anterior cingulate cortex (dACC) than non-T carriers^29^. In T carriers, the smaller dACC may lead to the lower performance on the attention/concentration index. These differences between the genotypes of *FKBP5* rs1360780 are likely to contribute to the differences in vulnerability to PTSD.

In consideration of the importance of the education years in the neurocognitive tests^30^, we confirmed that there was no significant difference in education years between the genotype groups not only among total individuals (Table 1), but also among young and aged individuals (data not shown).

Several limitations to this study need to be mentioned. First, its cross-sectional nature did not allow us to draw any definitive conclusions regarding age-dependent effects. Second, although random sampling would be desirable for collecting an unbiased representative sample, we recruited non-clinical volunteers from the community through local magazine advertisements and an announcement on our website. Third, although all participants were healthy subjects without a history of psychiatric disorders, they were not assessed for childhood trauma. Fourth, we performed ANCOVA without controlling for education years in the present study. However, essentially similar results were obtained in ANCOVA even when we controlled for education years, age, and gender (data not shown). Fifth, mood may be a confounder for attention/concentration, although we did not control for mood status. Further studies controlling for mood status are required to address this issue. Sixth, we assessed only Japanese mostly female subjects in the present study. Further studies in other ethnic groups are required to confirm our findings. We controlled for gender in ANCOVA. These limitations should be resolved in future studies.

In conclusion, we found that aged non-clinical individuals with the T allele of *FKBP5* rs1360780 had significantly poorer attention/concentration than those without. Since such cognitive dysfunctions are involved in PTSD symptoms, the results in our non-clinical population suggest that poor attention/concentration may be an endophenotype of individuals with this particular genetic vulnerability to PTSD. Our findings are of potential importance to provide new insights into the pathogenesis of stress-related psychiatric disorders, including PTSD, and may lead to the development of effective preventive strategies. Further studies are warranted to elucidate the mechanisms underlying this observed association.

## Methods

### Participants

Subjects were volunteers with no current or past history of psychiatric disorders. The number of subjects for the WMS-R and the WAIS-R were 742 and 627, respectively (Table 1). All subjects were biologically unrelated and Japanese. Participants were screened using the Japanese version of the Mini-International Neuropsychiatric Interview (M.I.N.I.)^31^,^32^ and unstructured interviews by a research psychiatrist. Individuals who had a prior medical history of central nervous system disease, substance abuse/dependence, severe head injury, dementia, or intellectual disability were not permitted to enroll in the study.

The present experiments on our participants were conducted in accordance with the Declaration of Helsinki. The study protocol was approved by the ethics committee of the National Center of Neurology and Psychiatry, Japan. After hearing a comprehensive description of the study, written informed consent was obtained from every subject.

### Genotyping

Venous blood was drawn from subjects and genomic DNA was extracted from whole blood according to standard procedures. The SNP rs1360780 was genotyped using the TaqMan 5′- exonuclease allelic discrimination assay (Applied Biosystems, Foster City, CA; assay ID C___8852038_10) as described previously^2^. PCR thermal cycling conditions were as follows: one cycle at 95°C for 10 min, followed by 50 cycles at 92°C for 15 s and 60°C for 1 min. Genotype data were assessed blinded to the case-control status. The genotyping protocol was performed according to our previous study^33^.

### Neurocognitive testing

To examine neurocognitive performance, the full Japanese versions of the WMS-R^34^,^35^ and WAIS-R^36^,^37^ were administered by research psychologists. The WMS-R measures me mory functions, namely verbal memory, visual memory, and delayed recall. In addition, it includes the attention/concentration index, which consists of the forward and backward digit and visual span subtests, thus measuring not only attention and concentration, but also verbal and spatial working memory. Auditory attention and verbal working memory were measured from the forward and backward digit span tests, respectively. Visual attention and visual working memory were measured from the forward and backward visual span tests, respectively. Of the 763 subjects, the WMS-R and WAIS-R were completed by 743 and 627 participants, respectively (Table 1). Among them, 607 ones overlapped in the WMS-R and WAIS-R test study groups.

### Statistical analysis

Deviation of genotype distributions from the HWE was assessed with a *χ^2^* test for goodness of fit. Demographic characteristics between genotype groups were compared by using either ANOVA or *χ^2^* test, as appropriate. Differences in WMS-R and WAIS-R scores between genotype groups were tested using an ANCOVA, controlling for age and gender. These tests were performed with SPSS ver.11 (SPSS Japan, Tokyo, Japan). Statistical tests were two-tailed and *P* values < 0.05 were considered significant.

## Author Contributions

T.F. designed the study, performed the genotyping, undertook the statistical analyses, and wrote the draft of the manuscript. J.M., Y.K., I.I. and A.N. administered the neuropsychological tests. H.H., M.O., K.H. and T.T., contributed to the data collection. H.K. organized recruitment of non-clinical volunteers, supervised the entire project, and gave critical comments on the manuscript. All authors contributed to and have approved the final manuscript.

## Figures and Tables

**Table 1:** Age, education years and gender distribution of subjects administered either the WMS-R or WAIS-R

	Genotype Groups				Statistics	*P* value
WMS-R	CC (*n* = 423)		CT/TT (*n* = 320)			
Mean age, years (SD)	45.1	(14.7)	43.3	(15.3)	*t* = 1.5, *df* = 741	0.13
Mean education years (SD)	15.1	(2.6)	15.0	(2.6)	*t* = 0.36, *df* = 741	0.72
Gender, female: *n* (%)	325	(76.8)	229	(71.6)	*χ^2^* = 2.7, *df* = 1	0.10
WAIS-R	CC (*n* = 360)		CT/TT (*n* = 267)			
Mean age, years (SD)	46.2	(14.4)	44.4	(16.0)	*t* = 1.4, *df* = 625	0.16
Mean education years (SD)	14.9	(2.7)	15.0	(2.6)	*t* = -0.073, *df* = 625	0.94
Gender, female: *n* (%)	276	(76.7)	192	(71.9)	*χ^2^* = 1.8, *df* = 1	0.18
